# Influencing factors of early childhood teachers’ disaster preparedness

**DOI:** 10.3389/fpubh.2023.1249736

**Published:** 2023-11-28

**Authors:** Young-Ran Lee, Sun-Nam Park, Mi-Ran Lee, Eunjeong Nam

**Affiliations:** ^1^Seoul Women's College of Nursing, Seoul, Republic of Korea; ^2^Yaedasom Childcare Center, Seoul, Republic of Korea

**Keywords:** early childhood, early childhood education and care, self-efficacy, resilience, COVID-19, teachers

## Abstract

**Background:**

The risk of disasters and infectious diseases continues to persist in modern times. Children are a vulnerable group in disaster prevention and management due to their limited ability to cope on their own. Hence, the role and disaster preparedness capacity of early childhood teachers (ECTs) is vital for children’s protection.

**Objectives:**

This study aims to explore how ECTs can improve their personal resilience to adapt to and overcome disasters as part of early childhood education and care (ECEC). To this end, this study examined the effects of ECTs’ self-efficacy, resilience, disaster awareness, COVID-19 stress, and work-related stress on their disaster preparedness.

**Results:**

According to the outcomes of disaster preparedness of ECTs based on their general and job characteristics, full-time employees and principals scored significantly higher in work-related disaster preparedness (WrDP) compared to part-time workers and general and assistant teachers, respectively. Resilience and WrDP were identified as influencing factors of general disaster preparedness (GdP), with an explanatory power of 26.4%. GdP and self-efficacy were identified as influencing factors of WrDP, with an explanatory power of 25.7%.

**Discussion:**

According to the findings, ECTs’ self-efficacy and GdP must be improved, followed by developing strategies to strengthen their resilience and WrDP. Doing so would ensure the safety and disaster preparedness of ECTs and infants who have low self-care capacity.

## Introduction

Disasters entail widespread losses and often exceed people’s capacity to cope with the aftermath. Depending on the severity and scope of disasters, they can involve human, material, economic, and environmental losses that require support at the global level (1). To minimize damages, disasters must be mitigated and recovery made possible through pre-preparation and appropriate responses (2).

When disaster prevention, preparedness, response, and recovery activities occur along a continuum, the negative impacts of a disaster are minimized; prevention and preparedness are the most cost-effective approaches in terms of invested manpower and resources (3, 4). Populations that are vulnerable to disaster prevention and response are high-risk groups whose safety and life support are threatened in the event of a calamity. Infants, children, older people, and people with disabilities are among the most in danger. In particular, infants and children lack self-management capacities owing to their lifecycle characteristics, and they therefore need efficient planning and strategies (5). In addition, children with poor connectivity are left with irreversible aftereffects in the event of a disaster, so effective preventative intervention is needed to enhance children’s connectedness (6).

Industrialization has altered the structure of the family, changing the family lifecycle and family functions. In South Korea, industrialization has led to the nuclearization of the family, a decrease in the number of family members per household, and recently, the expansion of various family types, such as single-parent families. Such adjustments are transforming the care and family functions of infants and young children within families.

The importance of early childhood education and care (ECEC) is increasing due to the growing number of children receiving care at child daycare centers. In this regard, the childcare activities and responsibilities of these centers are emphasized, and there is a growing interest in promoting the safety and health of infants and young children (7, 8). Given that infants and young children are a vulnerable group in terms of disaster awareness and response, early childhood teachers (ECTs) are an important resource when it comes to preventing disasters that could affect them (9) In disasters, ECTs complement the role of families, form bonds with children, and take responsibility for childcare activities and facilities (8). Through their role, ECTs can help children buffer the shock of disaster situations (6). In other words, being unprepared for a disaster has a negative impact on the well-being of ECTs as caregivers, which, in turn, affects children negatively. This can also be inferred from the Family Stress Model (10).

Disaster preparedness refers to all proactive planning and efforts that take place before a disaster strikes. ECTs’ awareness and competence in disaster preparedness not only allows them to protect themselves, but also infants and young children from the negative effects of disasters (6, 11, 12). Therefore, identifying factors that influence ECTs’ disaster preparedness lays the foundation for establishing effective strategies.

Infectious diseases are a social issue that has persisted since the beginning of humanity. In particular, the outbreaks and epidemics of severe acute respiratory syndrome (SARS) in the early 2000s, the Middle East respiratory syndrome (MERS) of 2015, and the COVID-19 pandemic and its variants since December 2019 have been—and still are—social disasters. The threat of various infectious diseases (including zoonotic diseases) continues to exist along with the possibility of natural catastrophes and the danger of multiple crises. Under such circumstances, resilience enables a person to adapt well in emergencies, and self-efficacy raises the efficiency of adaptation and response in stressful situations. Hence, the level of self-efficacy and resilience of ECTs are important factors in responding to and acting efficiently in stressful circumstances like disasters. However, the reality of disaster-related education and research concerning ECTs is that studies are limited to ECTs’ perceptions of disaster preparedness safety education (13), the relationship between infectious disease prevention knowledge and self-efficacy, emotional labor and infection prevention (14), ECTs’ COVID-19 situations and stress (15), as well as their perceptions and experiences and disaster preparedness (12). There is a lack of research that identifies the correlations between ECTs’ self-efficacy, resilience, stress, and disaster preparedness. As such, this study aims to determine the correlations between ECTs’ self-efficacy, resilience, disaster awareness, COVID-19 stress, and work-related stress. The study also intends to confirm the influencing factors of ECTs’ disaster preparedness and offer basic data that can be used to develop a program that improves ECTs’ disaster preparedness capacity. The availability of basic data will help to develop programs to build ECTs’ disaster management capacity, which in turn will benefit the health of infants, families, and communities.

## Methods

### Study design

This study has a descriptive design and was conducted to determine the impacts of ECTs’ self-efficacy, resilience, disaster awareness, COVID-19 stress, and work-related stress on their level of disaster preparedness.

### Participants

The participants of this study selected 7 out of 25 districts in Seoul and conducted convenience sampling to choose target daycare centers. The participants were ECTs working at public or *metropolitan* daycare centers in Seoul, South Korea, responsible for the care of children aged 3 to 7 years old. They understood the study’s objectives and gave written consent to take part in the survey. Using the G*Power (Version 3.1) program, the number of participants was based on a significance level of 0.05, a test power of 0.95, a moderate effect size of 0.15, and eight predictor variables. The calculated minimum sample size was 160, and considering a 10% dropout rate, data were collected from 176 participants. Excluding data from nine incomplete responses, data from 167 participants were analyzed.

### Measures

#### Self-efficacy

Self-efficacy was measured using Sherer et al.’s (16) tool modified by Kim (17). It has been modified and supplemented to align with the emotional aspects of Koreans, and it is frequently used to assess the specific self-efficacy of Korean adults. This tool inquiries about the level of confidence individuals have in their ability to successfully perform various activities, including daily life, and includes a self-efficacy variable related to problem-solving.

It is a 14-item tool with a scale that ranges from 1 for *not at all confident* to 10 for *completely confident*, with a minimum score of 14 and a maximum score of 140. Higher scores indicate a greater level of self-efficacy. In Kim’s (17) study, the reliability of the tool had a Cronbach’s α of 0.98, while in the present study, the Cronbach’s α was 0.96.

#### Resilience

Resilience was measured using the California Psychological Inventory (CPI) adapted by Klohnen (18) from the California Adult Q-Set (CAQ) by selecting 48 items that had a correlation of 0.20 or higher with items on self-resilience, and excluding one item that had a correlation with another CPI subscale. The final 29 items, translated by Park (19), were used. This tool was employed in a study that yielded results confirming the resilience of Korean. ECTs (20), and its statistical reliability and validity were verified, and its applicability to ECEC in Korea was confirmed. The tool used in this study consists of four subfactors—self-confidence (9 items), interpersonal effectiveness (8 items), optimism (10 items), and anger (2 items)—and each item is measured on a 5-point scale. Scores range from a minimum of 29 points to a maximum of 145 points, where a higher score indicate a greater level of self-resilience. In Park’s (19) study, the Cronbach’s α was 0.91, while in the present study, the Cronbach’s α was 0.92.

#### COVID-19 stress

The COVID-19 stress scale in this study involved all but one of the 21 items from the COVID-19 stress scale developed by Kim et al. (21). The original scale consists of three factors: Factor 1 is about fear of infection with 9 items, Factor 2 is about difficulty with social distancing with 6 items, and Factor 3 is about anger toward others with 6 items. This study used a modified version with 20 items that combined questions about rallies and gatherings under Factor 3. The reliability (Cronbach’s α) of Kim et al.’s (2021) tool was 0.914, while that of this study was 0.89.

#### Work-related stress

Work-related stress was measured using 23 items adapted from Lee et al. (7) to gage work-related stress in ECTs. These 23 items were taken from the 43 translated items (translated by the Korea Institute of Occupational Safety and Health), originally provided in the work-related stress measurement tool developed by the U.S. National Institute for Occupational Safety and Health (NIOSH). The tool includes a 4-point scale consisting of various sub-factors—job demands (4 items), job autonomy (4 items), interpersonal conflict (3 items), job insecurity (2 items), the organizational system (4 items), inadequate compensation (3 items), and work culture (3 items)—where a higher score implies a greater level of work-related stress. In Lee et al. (7), the reliability of the instrument had a Cronbach’s α of 0.90, while in the current study, the Cronbach’s α was 0.84.

### Disaster preparedness

#### General disaster preparedness

The tool used in this study to measure general disaster preparedness (GdP) was developed by the Japanese government to investigate adults’ general preparedness in the face of calamities (22). This study modified and supplemented 16 questions used by Han and Kwon (23), and added four questions on typhoons, traffic accidents, infectious diseases, and earthquakes, taking into account the frequency of natural catastrophes in South Korea. A total of 20 items on a scale ranging from 1 (*strongly disagree*) to 5 (*strongly agree*) were used, with a minimum score of 20 points and a maximum score of 100 points, where a higher score indicates a greater level of disaster preparedness. The Cronbach’s α of this study’s tool coincided with that of Han and Kwon’s (23) study at 0.83.

#### Work-related disaster preparedness

The work-related Disaster Preparedness Evaluation Tool (DPET) is currently widely used in Asia, including Japan and China (24–26). DPET is a disaster preparedness evaluation tool that classifies different phases of a crisis into the pre-disaster preparation phase, the disaster mitigation and response phase, and the disaster evaluation (recovery) phase (27); it consists of 46 questions. Among the 25 DPET items related to disaster preparedness translated by Han et al. (28), this study selected and revised 14 items relevant to ECTs and added one item on infectious diseases. Hence, a total of 15 questions were used for measurement. Each question was scored from 1 (*strongly disagree*) to 5 (*strongly agree*), with a total minimum score of 15 and a maximum score of 75, and a higher score indicating a greater level of disaster preparedness. The Cronbach’s α for each domain of the original DPET ranged from 0.91 to 0.93, while those of Han et al. (28) ranged from 0.94 to 0.96; that of this study was 0.92.

### Ethical considerations and data collection

The data were collected between October and December 2021 after receiving approval from the Institutional Review Board of the one of the authors’ institution (#202106-HR-010-03). The researcher gathered the data with the cooperation of 16 daycare centers that are licensed and operated as public or Seoul-based daycare centers. In accordance with the national social distancing policy, the researcher personally provided information on the study’s purpose and methods along with an explanation of the study to the directors, assistant directors, and nurses at the daycare centers. Afterward, the survey was administered to ECTs who gave written consent to participate. Participants were informed that they could discontinue the survey at any time, even after completion. The survey took about 20 min to complete.

### Data analysis

The collected data were analyzed using SPSS-win 21.0. The general and job-related characteristics of the ECTs were determined by frequency, percentage, mean, and standard deviation. Self-efficacy, resilience, COVID-19 stress, work-related stress, and disaster preparedness were established by mean and standard deviation. Differences in the level of disaster preparedness according to the general and job-related characteristics were analyzed through a t-test, analysis of variance, and a Scheffé test. Correlations between self-efficacy, resilience, COVID-19 stress, work-related stress, and disaster preparedness were examined using Pearson’s correlation coefficient, and factors affecting disaster preparedness were explored using stepwise multiple regression analysis.

## Results

### The general and job-related characteristics of the participants

In terms of the participants’ general characteristics, all were female, with an average age of 37.5 years (age range: 20–62), with 53 (32.7%) in the 20–29 age group, 43 (26.5%) in the 40–49 age group, 40 (24.7%) in the 30–39 age group, and 26 (16.1%) in the 50 and older age group. In terms of marital status, 82 (50.3%) were married. Regarding work-related characteristics, regarding certifications, 109 (66.1%) participants were Level 1 ECT-certified and 56 (33.9%) were Level 2 ECT-certified. Further, 126 (76.8%) were full-time employees, while 38 (23.2%) were part-time workers. By rank, 101 (60.8%) were general teachers, 36 (21.7%) were assistant teachers, 17 (10.3%) were head teachers, and 12 (7.2%) were directors. In addition, 56 (33.7%) had more than 10 years of on-the-job teaching experience, followed by 50 (30.1%) with 5–10 years of experience. Moreover, 138 (82.6%) had disaster preparedness training. The most common type of required disaster preparedness training was fire evacuation training with 85 respondents (57.4%), followed by infectious disease prevention training with 40 respondents (27.0%), and natural disaster (e.g., earthquakes and floods) evacuation training with 17 respondents (11.8%).

As for the types of support needed, 154 participants (92.2%) said they needed support from their national and local governments to improve preparedness and response to the pandemic, with 45 participants (29.2%) saying they needed vaccines and drugs, followed by 42 (27.3%) for personal protective equipment (PPE) and supplies, 31 (20.1%) for detailed guidance, and 25 (16.2%) for response training (see Table 1).

**Table 1:** Differences in disaster preparedness based on participants’ general and job-related characteristics.

(*N* = 167)						
		*n* (%)	General disaster preparedness		Work-related disaster preparedness	
			Mean ± SD	*t*/*F* (ρ)	Mean ± SD	*t*/*F* (ρ)
Age^†^ (year)	20–29	53 (32.7)	60.79 ± 10.16	1.47	56.57 ± 9.16	1.78
	30–39	40 (24.7)	59.40 ± 9.98	(0.224)	56.40 ± 9.68	(0.154)
	40–49	43 (26.6)	61.86 ± 9.04		59.81 ± 7.92	
	50 or older	26 (16.1)	64.31 ± 8.64		59.54 ± 7.82	
Marital status^†^	Married	82 (50.3)	60.61 ± 10.07	−0.93	57.48 ± 9.04	−0.78
	Single	81 (49.7)	62.04 ± 9.32	(0.349)	58.54 ± 8.47	(0.438)
Certification types^†^	Level 1 childhood educator	109 (66.1)	61.49 ± 9.26	−0.77	58.17 ± 8.38	−0.67
	Level 2 childhood educator	56 (33.9)	60.27 ± 10.18	(0.441)	57.21 ± 9.24	(0.502)
Employment types^†^	Full-time	126 (76.8)	61.69 ± 9.93	1.25	58.66 ± 8.75	2.08
	Part-time	38 (23.2)	59.42 ± 9.19	(0.211)	55.29 ± 8.70	(0.039)
Position^†^	Director	12 (7.2)	63.08 ± 10.00	5.27	65.33 ± 7.09^ab^	4.51
	Head teacher	17 (10.3)	60.24 ± 9.37	(0.845)	59.12 ± 8.31	(0.005)
	General teacher	101 (60.8)	61.25 ± 9.99		57.81 ± 8.48^a^	
	Assistant teacher	36 (21.7)	60.42 ± 9.45		55.06 ± 9.08^b^	
On-the-job teaching experience^†^ (years)	< 1	8 (4.8)	57.88 ± 16.44	0.29	54.25 ± 10.58	1.12
	1 ≤, < 3	31 (18.7)	61.52 ± 9.22	(0.883)	57.42 ± 9.12	(0.350)
	3 ≤, <5	21 (12.7)	60.57 ± 8.96		56.33 ± 9.70	
	5 ≤, < 10	50 (30.1)	60.96 ± 9.72		57.56 ± 8.13	
	≤ 10	56 (33.7)	61.66 ± 9.44		59.64 ± 8.40	
Experience in disaster preparedness education	Yes	138 (82.6)	61.38 ± 9.71	0.81	57.97 ± 8.76	0.39
	No	29 (17.4)	59.76 ± 9.85	(0.417)	57.28 ± 8.95	(0.699)
Need for disaster preparedness education	Yes	148 (88.6)	61.03 ± 9.79	−0.26	57.39 ± 8.81	
	No	19 (11.4)	61.63 ± 9.43	(0.799)	61.47 ± 7.74	
Types of necessary disaster preparedness education	Fires	85 (59.0)				
	Natural disasters	17 (11.8)				
	Maritime accidents	2 (1.4)				
	Infectious diseases	40 (27.8)				
Necessary government support	Yes	154 (92.2)	61.22 ± 9.89	0.57	57.60 ± 8.78	−1.29
	No	13 (7.8)	59.62 ± 7.60	(0.569)	60.85 ± 8.38	(0.200)
	Education	11 (7.2)				
	Response training	25 (16.2)				
	Equipment, supplies	42 (27.3)				
	Vaccines, drugs	45 (29.2)				
	Detailed guidance	31 (20.1)				

† Included missing data.

ab Scheffe *t*-test.

### Self-efficacy, resilience, work-related stress, COVID-19 stress, and disaster preparedness of ECTs

The score for self-efficacy averaged 105.75 ± 19.18 points out of 140 (61–140 points). The score for resilience averaged 109.93 ± 16.50 points out of 145 (69–145 points), with its sub-factors displaying the following scores: self-confidence at 34.54 ± 5.76 points (19–45 points), interpersonal effectiveness at 29.46 ± 6.17 points (14–40 points), optimism at 38.91 ± 5.97 points (23–50 points), and anger management at 7.02 ± 1.88 points (3–10 points). ECTs suffered from COVID-19 stress and work-related stress. The score for COVID-19 stress averaged 27.28 ± 10.90 points out of 80 (4–60 points), with its sub-factors demonstrating the following scores: fear of infection at 10.70 ± 6.73 points (0–33 points), difficulty with social distancing at 12.29 ± 4.57 points (0–24 points), and anger toward others at 4.29 ± 3.39 points (0–15 points). The score for work-related stress averaged 49.84 ± 7.94 points (28–76 points) out of 92, with its sub-factors displaying the following scores: job demands at 10.82 ± 2.64 points (4–23 points), job autonomy at 10.02 ± 1.31 points (6–15 points), relationship conflicts at 5.76 ± 2.14 points (3–27 points), job insecurity at 3.70 ± 1.40 points (2–8 points), the organizational system at 7.60 ± 1.78 points (3–13 points), inadequate compensation at 6.23 ± 1.33 points (3–10 points), and workplace culture at 5.71 ± 1.69 points (2–10 points).

The degree of disaster preparedness was classified into general and work-related disaster preparedness (WrDP). The score for GdP averaged 61.10 ± 9. Seventy two points out of 100 (41–98 points), and that for WrDP averaged 57.85 ± 8.77 points out of 75 (28–75 points), with its sub-factors demonstrating the following scores: the preparation phase at 25.46 ± 3.45 points (13–30 points), the response phase at 13.24 ± 3.35 points (4–20 points), and the evaluation (recovery) phase at 19.15 ± 3.26 points (11–25 points) (see Table 2).

**Table 2:** The degree of self-efficacy, resilience, work-related stress, COVID-19 stress, and disaster preparedness of ECTs.

(*N* = 167)				
		Mean ± SD	Minimum	Maximum
Self-efficacy		105.75 ± 19.18	61.0	140.0
Resilience		109.93 ± 16.50	69.0	145.0
	Self-confidence	34.54 ± 5.76	19.0	45.0
	Interpersonal effectiveness	29.46 ± 6.17	14.0	40.0
	Optimism	38.91 ± 5.97	23.0	50.0
	Anger management	7.02 ± 1.88	3.0	10.0
COVID-19 stress		27.28 ± 10.90	4.0	60.0
	Fear of infection	10.70 ± 6.73	0.0	33.0
	Difficulty with social distancing	12.29 ± 4.57	0.0	24.0
	Anger toward others	4.29 ± 3.39	0.0	15.0
Work-related stressors		49.84 ± 7.94	28.0	76.0
	Job demands	10.82 ± 2.64	4.0	23.0
	Job autonomy	10.02 ± 1.31	6.0	15.0
	Relationship conflicts	5.76 ± 2.14	3.0	27.0
	Job insecurity	3.70 ± 1.40	2.0	8.0
	Organizational system	7.60 ± 1.78	3.0	13.0
	Inadequate compensation	6.23 ± 1.33	3.0	10.0
	Workplace culture	5.71 ± 1.69	2.0	10.0
General disaster preparedness		61.10 ± 9.72	41.0	98.0
Work-related disaster preparedness		57.85 ± 8.77	28.0	75.0
	Preparation phase	25.46 ± 3.45	13.0	30.0
	Response phase	13.24 ± 3.35	4.0	20.0
	Evaluation phase	19.15 ± 3.26	11.0	25.0

### Differences in disaster preparedness among ECTs according to their general and job-related characteristics

There were no significant differences in GdP according to the general and work-related characteristics of ECTs. There were, however, significant differences in WrDP with respect to employment type and rank. Full-time teachers scored an average of 58.66 ± 8.75 points, which was significantly higher than part-time teachers, who scored 55.29 ± 8.70 points (*t* = 2.08, *p* = 0.039). Moreover, directors of childcare centers scored an average of 65.33 ± 7.09 points, which was significantly higher than the scores of general teachers at 57.81 ± 8.48 points and assistant teachers at 55.06 ± 8.08 points (*F* = 4.51, *p* = 0.005) (see Table 1).

### Correlations between disaster preparedness and ECTs’ self-efficacy, resilience, disaster awareness, COVID-19 stress, and work-related stress

This study examined the correlations between disaster preparedness and ECTs’ self-efficacy, resilience, disaster awareness, COVID-19 stress, and work-related stress, and found that GdP was positively associated with higher self-efficacy (*r* = 0.220, *p* = 0.004), stronger resilience (*r* = 0.352, *p* < 0.001), and lower work-related stress (*r* = −0.249, *p* = 0.001). WrDP was associated with higher self-efficacy (*r* = 0.309, *p* < 0.001), stronger resilience (*r* = 0.286, *p* < 0.001), lower work-related stress (*r* = − 0.209, *p* = 0.007), and increased GdP (*r* = 0.471, *p* < 0.001) (see Table 3).

**Table 3:** Correlations between disaster preparedness and ECTs’ variables.

(*N* = 167)						
	Self-efficacy	Resilience	Disaster awareness	COVID-19 stress	Work-related stressors	General disaster preparedness
*r*(*p*)						
General disaster preparedness	0.220	0.352	0.146	0.007	−0.249	
	(0.004)	(< 0.001)	(0.059)	(0.924)	(0.001)	
Work-related disaster preparedness	0.309	0.286	0.142	−0.113	−0.209	0.471
	(< 0.001)	(< 0.001)	(0.066)	(0.147)	(0.007)	(< 0.001)

### Factors influencing the disaster preparedness of ECTs

A stepwise multiple regression analysis was performed on the variables of self-efficacy, resilience, work-related stress, and WrDP—which were determined to be correlated to ECTs’ GdP—to identify the factors influencing GdP. According to analysis, WrDP and resilience were found to be influencing factors with an explanatory power of 26.4% (*F* = 30.76, *p* = <0.001) (see Table 4). Upon verifying the assumptions of the independent variables in the multiple regression analysis, the tolerance limit was determined to be 0.918 (less than 1.0) and the variation inflation factor (VIF) was 1.089 (less than 10), demonstrating no multicollinearity problems. Furthermore, the Durbin-Watson value was 1.727, indicating that the autocorrelation of errors was mutually independent.

**Table 4:** Influencing factors of general disaster preparedness of ECTs.

(*N* = 167)								
	*B*	*S.E*	*β*	*t(ρ)*		Adj *R*^2^	*F*	*ρ*
	19.94	5.39		3.70	(< 0.001)	0.264	30.76	< 0.001
Work-related disaster preparedness	0.45	0.08	0.40	5.80	(< 0.001)			
Resilience	0.14	0.04	0.24	3.40	(0.001)			

Likewise, a stepwise multiple regression analysis was carried out on the variables of self-efficacy, resilience, work-related stress, and GdP—all of which were correlated to ECTs’ WrDP—in order to identify the factors influencing WrDP. According to the analysis, disaster preparedness and self-efficacy were influencing factors with an explanatory power of 25.7% (*F* = 29.71, *p* = <0.001). When checking the assumptions of the independent variables in the multiple regression analysis, the tolerance limit was determined to be 0.952 (less than 1.0) and the VIF was 1.051 (less than 10), demonstrating no multicollinearity problems. Moreover, the Durbin-Watson value was 1.681, indicating that the autocorrelation of errors was mutually independent (see Table 5).

**Table 5:** Influencing factors of work-related disaster preparedness of ECTs.

(*N* = 167)								
Independent variables	*B*	*S.E*	*β*	*t(ρ)*		Adj *R*^2^	*F*	*ρ*
	24.08	4.48		5.37	(< 0.001)	0.257	29.71	< 0.001
General disaster preparedness	0.38	0.06	0.42	6.17	(< 0.001)			
Self-efficacy	0.10	0.03	0.22	3.16	(0.002)			

## Discussion

COVID-19 has profoundly impacted individuals and workplaces, but its impact on ECTs is unclear (29). The pandemic has influenced ECTs’ work practices, leading to work-related stress and burnout (30). In response, multidimensional efforts are recommended to increase understanding of work-related stress and promote resilience. This research was conducted as a preliminary study to identify the influencing factors for ECTs when adapting to and overcoming disaster situations as part of carrying out ECEC, while also providing measures to promote individual resilience.

To this end, the study classified factors that affect ECTs’ disaster preparedness into two main areas, and discussed problems identified during the analysis.

First, through the examination of disaster preparedness based on general and job characteristics, it was found that there were differences in the degree of preparedness depending on employment type and rank. Full-time teachers and directors displayed higher WrDP than part-time teachers and general or assistant teachers, respectively. This finding is consistent with that of previous research, which suggests that less experienced educators are less capable of managing adversity experienced by children compared to their more experienced counterparts (31). The sudden onset of COVID-19 left ECTs unprepared in both personal and professional domains (32), and has led to a significant spike in ECTs’ workload due the high likelihood of infections in the workplace, increased number of tasks, and greater responsibility regarding children’s safety (32). Under such circumstances, full-time teachers with relatively higher work stability and directors with more experience may have been able to adapt more easily to sudden changes that followed the COVID-19 pandemic. Disaster management capacity is the ability to respond immediately to disasters and minimize damage, and it is essential for ECTs, who need to respond promptly to calamities and reduce children’s and their own vulnerability to hazards (7). It is believed that groups with a fairly high level of stability and more experience manifest greater adaptability by setting goals and planning in stressful situations.

Second, resilience and WrDP were identified as influencing factors of ECTs’ GdP, with an explanatory power of 26.4%. A study of Italian teachers found that those with lower baseline resilience experienced more COVID-19-related symptoms of anxiety, depression, stress, and burden than those with higher resilience (33). Self-resilience refers to the ability to respond appropriately in unfamiliar situations, and it is believed that resilience acts as a force that enables people to flexibly cope with diverse stressful circumstances, thereby minimizing physical and mental symptoms. Furthermore, self-efficacy and GdP were identified as influencing factors of ECTs’ WrDP, with an explanatory power of 25.7%. This finding is consistent with that of a previous study (12), which determined that greater self-efficacy and GdP among ECTs revealed a higher level of WrDP. Resilience and self-efficacy are beliefs in one’s ability to successfully perform a task, and they are deemed to act as moderating factors in improving disaster management capabilities.

Third, a lack of institutional support that encourages resilience and self-efficacy prevents the implementation of practical capacity-building training. Tension caused by the COVID-19 pandemic has had a significant impact on the emotional well-being and mental health of ECTs (34), acting as a major stressor. Compared to a preceding study conducted with the same ECTs in 2021 (12), there was a slight increase in resilience, but a dip in self-efficacy. As the pandemic became prolonged, the resilience of ECTs—who had gotten used to changing circumstances—increased. Nonetheless, their sense of self-efficacy declined somewhat because they felt inadequate when responding to disasters and experienced difficulties in their disaster response capabilities in comparison to the beginning of the COVID-19 pandemic. The reasons behind such reactions may be inferred from Quinones (35). Some ECTs perceived that most disaster response manuals were structured around schoolteachers, and that their work was not as worthy and that they were treated with less value than schoolteachers (35). This may have served as an extrinsic motivation for ECTs in lowering their self-belief during emergency situations, which in turn contributed to lower self-efficacy. The present study confirmed that most ECTs in such a scenario are in need of disaster preparedness training and active forms of state support. In addition to human and material resources for disaster preparedness, these teachers also need detailed guidelines for disaster response.

While the emotional health and well-being of infants and young children during COVID-19 have been discussed (36), there has been a lack of research on the health and protection of ECTs, who are responsible for the care and education of these children (37). According to this study, when ECTs’ emotional health and wellbeing are given higher priority than those of infants and young children, quality care and education will be provided, and their disaster response capacity will also increase. Hence, disaster response capacity training that strengthens the self-efficacy and resilience of these teachers should be implemented. This study also asserts that increasing disaster response capacity is not limited to the health and safety of infants and young children; it also plays a crucial role in managing the health of the general population through the national infection control system. In this regard, modifications should be made to the current direction of education and mental health promotion of disaster response in South Korea, which focuses on students and young children. The Ministry of Health and Welfare and related organizations have prepared disaster response manuals and psychological support plans for infants and young children and distributed them to frontline organizations. The major issue, however, is that there is an absence of protection and capacity-building support policies for ECTs, who protect and educate these children in difficult situations. As such, this study calls for active training and support to enhance the self-efficacy and resilience of ECTs working in ECEC settings to reduce disaster stress. This study highlights the necessity of stockpiling emergency medications, as well as the provision of PPE and supplies requested by those in ECEC settings, in addition to strengthening disaster response capacity at schools. The CDC recommends tailored guidance for teachers of young children (38). In line with this advice, this study calls for the development of specific disaster response guidelines for ECTs working with infants and toddlers in ECEC settings, as well as enhanced on-site response drills that may be practiced in the presence of infants and toddlers. Strategies for disaster preparedness capacity building should be tied to community settings, such as the availability of resources, the health status of infants and toddlers, and the community’s demographic composition (39). Enhanced prevention strategies may also be necessary in ECCE settings (39).

The participants in this study were selected using convenience sampling and ECTs working in Seoul, South Korea, which has a relatively well-established disaster response system, so the results cannot be said to represent the situations of all ECTs across the country. Hence, the findings cannot be generalized, and future research should be conducted by recruiting participants through nationwide random sampling. Moreover, this study is limited in that it used self-reported data based on a cross-sectional survey design, which makes it difficult to identify a causal relationship between the variables. In addition, unexamined variables were not controlled for.

Future research should collect qualitative data through focus group interviews and quantitative studies should be performed that include diverse variables to derive comprehensive impact variables in order to not only examine disaster preparedness from an ecological standpoint, but also look more closely into the influencing factors to specifically identify responses at the individual, community, and national levels. Nevertheless, this study is meaningful in that it calls attention to ECTs, who are the caregivers of infants and toddlers and manage their health in the context of South Korea, where disaster response training and healthcare are carried out at childcare centers. In particular, it is significant because it offers an opportunity to reevaluate the limitations of disaster response management after a disaster by conveniently selecting and confirming the capital city that is best managed by the country. Based on the outcomes, this study also explored ways to strengthen ECTs’ disaster response capacity.

## Conclusion

The findings stress the importance of actively providing education and support to increase self-efficacy and resilience in individual ECTs in order to strengthen their capacity to respond to disasters. They also highlight the significance of having numerous discussions about lowering disaster-related stress. The education and mental health promotion policies for disaster response in South Korea, which are currently only implemented for infants and toddlers, must also include ECTs.

## Data availability statement

The raw data supporting the conclusions of this article will be made available by the authors, without undue reservation.

## Ethics statement

The studies involving humans were approved by the Institutional Review Board of the Seoul Women’s College of Nursing (#202106-HR-010-03). The studies were conducted in accordance with the local legislation and institutional requirements. The participants provided their written informed consent to participate in this study.

## Author contributions

All authors contributed to the study conception and design. Material preparation, data collection, and analysis were performed by Y-RL, S-NP, M-RL, and EN. The first draft of the manuscript was written by Y-RL and all authors commented on previous versions of the manuscript. All authors read and approved the final manuscript. All authors contributed to the article and approved the submitted version.
